# The dynamic role of the ilio-sacral joint in jumping frogs

**DOI:** 10.1098/rsbl.2018.0367

**Published:** 2018-09-12

**Authors:** Christopher T. Richards, Enrico A. Eberhard, Amber J. Collings

**Affiliations:** 1 The Royal Veterinary College, Hawkshead Lane, Hatfield AL9 7TA, UK; 2 Institute of Criminal Justice Studies, University of Portsmouth, Museum Road, Portsmouth PO1 2QQ, UK

**Keywords:** frogs, jumping, kinematics, inverse dynamics, pelvis

## Abstract

A striking feature among jumping frogs is a sharp pelvic bend about the ilio-sacral (IS) joint, unique to anurans. Although this sagittal plane hinge has been interpreted as crucial for the evolution of jumping, its mechanical contribution has not been quantified. Using a model based on *Kassina maculata* and animated with kinematics from prior experiments, we solved the ground contact dynamics in MuJoCo enabling inverse dynamics without force plate measurements. We altered the magnitude, speed and direction of IS extension (leaving remaining kinematics unaltered) to determine its role in jumping. Ground reaction forces (GRFs) matched recorded data. Prior work postulated that IS rotation facilitates jumping by aligning the torso with the GRF. However, our simulations revealed that static torso orientation has little effect on GRF due to the close proximity of the IS joint with the COM, failing to support the ‘torso alignment’ hypothesis. Rather than a postural role, IS rotation has a dynamic function whereby angular acceleration (i) influences GRF direction to modulate jump direction and (ii) increases joint loading, particularly at the ankle and knee, perhaps increasing tendon elastic energy storage early in jumps. Findings suggest that the pelvic hinge mechanism is not obligatory for jumping, but rather crucial for the fine tuning of jump trajectory, particularly in complex habitats.

## 1. Introduction

Anuran anatomy is unique among vertebrates, owing in part to the elongations and reorientations of pelvic bones during their evolutionary transition from salamander-like tetrapods [1]. Morphologists have long recognized the pelvis as a crucial bio-mechanical apparatus facilitating not only jumping [2], but also walking [2,3] and swimming [4]. In particular, a novel hinge at the ilio-sacral (IS) joint is a hallmark of jumping species allowing extension in the sagittal plane to straighten the back, aligning the hindlimb ground reaction force (GRF) with the torso [2]. ‘Sagittal-hinge’ jumpers arose multiple times independently [3] hinting that fossil presence of the IS joint is evidence of hopping early in frog evolution [3,5].

To elucidate the role of IS extension, electromyography [2,6], cineradiography [5,7] and inverse dynamics (ID; [7]) have been used to determine that pelvic muscles activate synchronously early in jumps causing IS extensor torque to drive rapid extension of the back. Although the above studies show neuro-mechanical activity of the IS joint, no analysis has quantified its direct effect on centre of mass (COM) mechanics, joint torques or GRF.

Does increased pelvic rotation in the sagittal plane increase jump distance? We hypothesize that increased IS extension will (i) reorient the body axis in line with the GRF to prevent torque about the COM [2,5] and (ii) reorient the GRF to influence jump direction. H1 implies that IS rotation has a static postural effect which should influence GRF regardless of torso angular velocity. In contrast, H2 predicts that angular IS acceleration is more crucial, incurring counterbalancing leg torques through inertial coupling of the segments [2] and redirecting GRF to influence COM direction of travel. To test H1 and H2, we developed a novel modelling approach allowing us to simulate the impact of pelvic rotation on jumping by calculating joint torques and GRF in response to manipulated frog jump kinematics.

## 2. Material and methods

**Figure 1.**
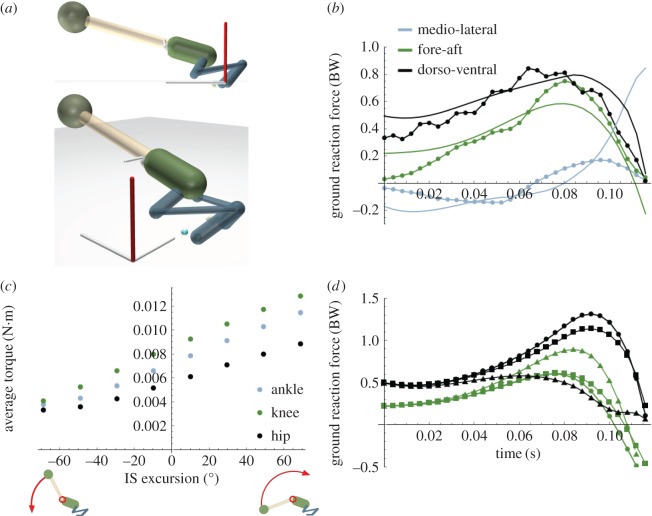
Simulating frog jumping. (*a*) Side/top views of the model (electronic supplementary material, table S1) with legs (blue), pelvis (green), spine (white) and head (sphere) with diameters to insure correct segment masses. The head is a point mass giving the appropriate torso moment of inertia. The white rod represents the IS hinge axis (0° = horizontal; +90° = vertical). The global reference frame is shown with the *Z*-axis (red). (*b*) Comparison of simulated (solid) versus measured (dotted) ground reaction force (GRF) components in lateral+/medial−(blue), fore+/aft−(green) and dorsal+/ventral−(black) for the duration of ground contact (final time = take-off). (*c*) Average joint torque magnitude versus IS angle excursion (=final angle − initial angle). Negative excursions are downward rotation (flexion). (*d*). GRF from three example jumps: steepest (circle), farthest (square) and shortest-most horizontal (triangle). Lateral/medial force omitted for clarity.

A 3D ‘rig’ was created in MuJoCo [8] (figure 1*a*; electronic supplementary material, table S1) using mass-inertia quantified from contrast-enhanced μCT images of *Kassina maculata*, a walker–jumper with jumping abilities comparable to other groups such as ranids [9]. To base our simulations, we used an example jump (∼median take-off angle) from experiments [9]. Left leg kinematics were mirrored to create right leg kinematics. Data were converted to unit quaternions, and smoothed using Hopf coordinates (electronic supplementary material, appendix S1).

We modified the nominal jump by manipulating IS angle while holding the remaining kinematics consistent. We used a sigmoidal (rapidly accelerating) function to flex/extend the IS joint (electronic supplementary material, figure S1).

Kinematics were imported into custom software using the MuJoCo C++ library. We performed ID with simulated contact (IDC) to compute GRF and joint torques (electronic supplementary material, appendix S3). ID without force measurements is possible with MuJoCo's innovation of mathematically invertible dynamics via a ‘soft contact’ model [8]. This approach differs from forward dynamics which iteratively refine joint torques until the simulation converges on prescribed kinematics. Despite their differences, both approaches share the goal of deriving torques for given kinematics.

Each simulation was run until the vertical GRF crossed zero, indicating take-off, after which the frog COM was modelled ballistically to estimate jump distance [10]. Because the ground contact point was defined as the proximal end of the tarsals, our simulations do not account for time-varying contact as the foot peels off of the substrate during jumps [9], thus jump distance is restricted compared to *in vivo* jumps because the foot is effectively ‘glued’ to the substrate.

We validated our analysis and model predictions by comparing simulated GRF data with experimentally recorded jumping performance [9].

## 3. Results and discussion

Using inverse dynamics contact analysis (IDC; electronic supplementary material, movies S1–S3) we determined the hypothetical role of pelvic rotation about the ilio-sacral (IS) joint to test whether extension in the sagittal plane enhances jump performance as postulated [2,5]. Even in the absence of measurements of bone rotations (e.g. [7]), experimentally recorded versus simulated GRF from IDC showed similar patterns and magnitudes (error ∼±0.1 BW) except for two notable discrepancies (figure 1*b*). Our model does not include thrust from the arms [11], accounting for larger error (±0.2 BW) during early push-off. Hence, the model overestimated both forward and vertical GRF early in the jump. Additionally, medio-lateral forces were consistent in their pattern of shifting from medial force to lateral force throughout the jump, yet were exaggerated in our model. This inaccuracy was ultimately caused by left–right asymmetry during recorded jumps. Because right kinematics were mirrored from the left, the ‘extra’ medio-lateral force can be interpreted as the additional force required to impose symmetry.

### (a) Ilio-sacral rotation influences joint torque and ground reaction force

Upwards torso rotation was simulated by a low initial angle (flexed IS; torso closer to the ground), ending in higher angles. Modulating the initial versus final IS angle created a range spanning from downward rotation (flexion; negative IS excursion) to fixed IS angle (IS excursion = 0) to upward rotation (extension; positive IS excursion). Because of the increased kinetic energy to rotate the torso, greater IS extension increased GRF magnitude thereby increasing joint torque, particularly at the ankle and knee (figure 1*c*; table 1).

**Table 1.** Summary data.

	nonlinear IS extension (sigmoidal increase in IS angle; angular acceleration > 0) See electronic supplementary material, figure S1			linear IS extension (i.e. angular acceleration = 0) See electronic supplementary material, figure S2		
summary data pooled over all simulation conditions	min	max	range	min	max	range
vertical impulse (N·s)	0.012	0.022	0.01	0.016	0.017	0.001
horizontal impulse (N·s)	0.008	0.013	0.005	0.009	0.01	0.001
jump distance (body lengths)	0.0	0.891	0.891	0.0	0.51	0.51
take-off angle (°)	1.4	51.6	50.2	17.3	37.7	20.4
mean hip torque magnitude (N·m )	0.003	0.009	0.006	0.004	0.006	0.002
mean knee torque magnitude (N·m )	0.004	0.013	0.009	0.007	0.009	0.002
mean ankle torque magnitude (N·m )	0.004	0.011	0.007	0.005	0.008	0.003
peak resultant GRF (N)	0.222	0.338	0.116	0.229	0.241	0.012

**Figure 2.**
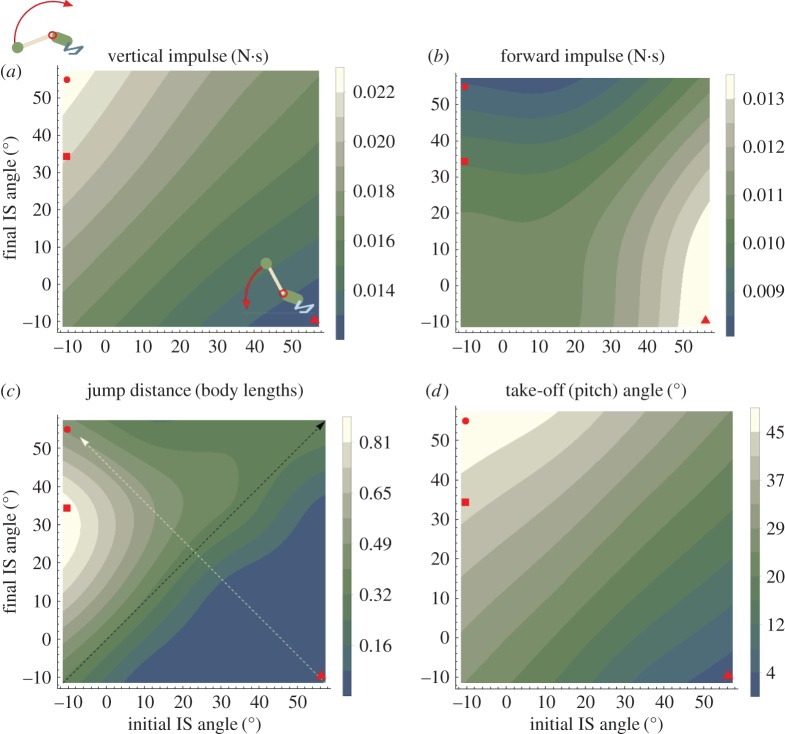
Mapping the effect of IS extension versus flexion (upper left versus lower right regions, respectively). (*a*) Vertical impulse, (*b*) horizontal impulse, (*c*) jump distance, (*d*) take-off angle. The black arrow (in *c*, but applies to *a*–*d*) represents increasing fixed torso angles (IS angular velocity = 0). The white arrow represents flexion to extension (as in figure 1*c*). Red symbols mark the steepest (circle), farthest (square) and shortest (triangle) example jumps.

Upward rotation increased vertical (dorsoventral) GRF at the expense of horizontal force (figure 1*d*), whereas downward rotation caused increased horizontal, but lower vertical GRF. Consequently, upward rotating simulations produced greater vertical than horizontal impulse (figure 2*a*,*b*; red circle versus triangle) resulting in take-off (pitch) angles approaching approximately 45° enabling farther jumps (figure 2*c*,*d*). Hence, IS extension can not only modulate dorsoventral force and take-off angle, but also may influence ground contact duration by advancing or delaying when vertical GRF crosses zero (electronic supplementary material, movie S4).

### (b) Ilio-sacral rotation is dependent on acceleration, not posture

We expected that IS joint rotation would enhance jumping performance through postural alignment of the torso with the GRF (H1) and by creating counterbalancing GRF to influence take-off angle (H2). Simulated data support H2, but not H1. The clearest evidence countering H1 are simulations where IS angle was fixed within trials, but increased between trials to hypothetically simulate torso pre-alignment prior to jumps (electronic supplementary material, movie S5). These static IS trials are shown along the upward-right diagonal of performance maps (figure 2). Across all performance metrics, there was no effect of static IS angle posture. Additionally, all effects of pelvic rotation disappeared when IS rotational velocity was held constant (table 1). Together, these observations support that frogs need not align their torso with the GRF [5] simply because torso orientation does not greatly influence the COM location. Rather than aiming the torso, frogs realign the GRF itself which is achieved by torso rotational acceleration, perhaps analogous to swinging ‘halteres’ in human sporting [12]. The muted effect of static torso orientation is due to the close proximity of the COM to the hinge axis itself, which we postulate may have a stabilizing role after take-off (see below).

### (c) Simulation results predict that the pelvic mechanism helps to pre-load tendons for enhanced elastic energy storage and modulates jump direction

Aside from transmitting force from hindlimbs to the torso [5], our simulations reveal two additional putative roles for the pelvic mechanism. Firstly, during early launch the forelimbs produce considerable vertical force [11] helping to extend the IS joint before the arms lift off [5]. This early IS extension produces higher joint torques and muscle forces, particularly at the ankle and knee (figure 1*c*; table 1). At the ankle, frogs likely use a catch-release mechanism to provide initial resistance required for muscle to stretch elastic tendons followed by explosive recoil upon catch release [13] mediated by a shift from low-to-high limb mechanical advantage [7,14,15]. Assisting this ‘inertial catch’, we propose upwards rotational acceleration of the torso further enhances jumps by increasing muscle loading (via increased joint torque) for greater stretching. Moreover, frogs may modulate IS kinematics either to advance or delay peak muscle force to fine tune the timing of elastic recoil. Importantly, there are no tendons in our model, therefore future forward dynamics analysis would be required to determine whether IS-assisted preloading causes farther jumps. Secondly, the close anatomical proximity of the COM to the IS joint is potentially important for influencing whole-body angular velocity. Because rigid bodies tumble about their COM, frogs can potentially change their angular momentum via subtle IS accelerations to fine tune pitch for steepness (dorsoventral pelvic rotation) or yaw for turning (lateral pelvic rotation) with minimal need to impart linear momentum of the COM. Hypothetically, this angular momentum control could be crucial for navigating to perches for arboreal frogs.

### (d) Simulations predict *in vivo* behaviour

Consistent with simulations, peak IS accelerations recorded from experiments [9] strongly predict increases in joint torque and vertical GRF (table 2; electronic supplementary material).

**Table 2.** IS kinematics versus performance for *N* = 50 *in vivo* jumps (see electronic supplementary material). Parameters are from a general linear model run using LinearModelFit in Mathematica 10 (Wolfram Research, Champaign, IL, USA).

independent variable (A)	dependent variable (B)	*p*-value A versus B	significance at *p* = 0.05	*p*-value frog	significance at *p* = 0.05	*p*-value A*frog	significance at *p* = 0.05
max IS angular acceleration	take-off angle	≪0.01	Y	0.789401	N	0.901176	N
IS angular excursion	take-off angle	≪0.01	Y	0.788246	N	0.0680066	N
max IS angular acceleration	peak vertical GRF	≪0.01	Y	0.589809	N	0.919585	N
max IS angular acceleration	peak horizontal GRF	0.112597	N	0.00346813	Y	0.106532	N
max IS angular acceleration	mean hip torque	0.00773526	Y	0.0000240658	Y	0.21468	N
max IS angular acceleration	mean knee torque	≪0.01	Y	0.0807289	N	0.173157	N
max IS angular acceleration	mean ankle torque	≪0.01	Y	≪0.01	Y	0.042	Y

## 4. Conclusion

To overcome challenges of studying the isolated effects of IS rotation, we performed inverse dynamics from simulated kinematics. Simulations suggest that IS rotation, via the uniquely jointed anuran pelvis, enhances jumping by helping modulate COM trajectory and loading the ankle for greater elastic energy storage-recoil. Hence, IS mobility is likely important among the suite of saltatorial features including elongated hindlimbs [1] and powerful muscle–tendon systems [14]. Further work using XROMM and musculoskeletal modelling could explore how pelvic musculature of specialized jumpers produces power for tightly timed IS acceleration to modulate take-off/flight trajectory. In contrast to prior interpretations [5], we propose IS rotation is most important for enhancing jump performance and control, but is not obligatory for torso–GRF alignment. When comparing ancient proto-frogs with progressively modern fossils, derived hindlimb features predate the development of the mobile IS joint complex [1]. This fossil evidence, combined with our simulated data, is consistent with the interpretation that early frogs were adept hoppers, but not necessarily great leapers like modern anurans [3].

## Supplementary Material

slerpID_BL_sub01_SIappendices.docx

## Supplementary Material

SIDATA_linear.xlsx

## Supplementary Material

SIDATA_nonlinear.xlsx

## Supplementary Material

invivoDATA.xlsx

## Supplementary Material

SI movie 1

## Supplementary Material

SI movie 2

## Supplementary Material

SI movie 3

## Supplementary Material

SI movie 4

## Supplementary Material

SI movie 5

## Data accessibility

Raw simulation data are provided in the electronic supplementary material.
