# Supplementary material for: The dynamic role of the ilio-sacral joint in jumping frogs
Source: Biol Lett. 2018 Sep 12;14(9):20180367. doi: 10.1098/rsbl.2018.0367 (PMC6170761; doi:10.1098/rsbl.2018.0367)
Supplement: slerpID_BL_sub01_SIappendices.docx [file rsbl20180367supp1.docx]

SUPPLEMENTARY INFORMATION

Supplementary files included:

| Filename | File type | Data content |
| --- | --- | --- |
| invivoDATA.xlsx | Spreadsheet | Performance data recorded from in vivo jumps |
| SIDATA_linear.xlsx | Spreadsheet | Inverse dynamics and kinematics time-series data output from 16 simulations using a linearly increasing IS angle. |
| SIDATA_nonlinear.xlsx | Spreadsheet | Inverse dynamics and kinematics time-series data output from 16 simulations using a sigmoidally increasing IS angle. |
| slerpID_SImov1.mov | Video | Please see captions below |
| slerpID_SImov2.mov | Video | Please see captions below |
| slerpID_SImov3.mov | Video | Please see captions below |
| slerpID_SImov4.mov | Video | Please see captions below |
| slerpID_SImov5.mov | Video | Please see captions below |

Table S1. Rig morphology and local reference frames

| Simplified segments (rounded cylinders) | Mass (g) | Parent reference frame | Joint | Joint degrees of freedom | Additional joint constraints |
| --- | --- | --- | --- | --- | --- |
| Left foot | 0.012 | Ground | ”free joint”: position/orientation with respect to ground | 6 | None |
| Left tarsals | 1.145 | Left foot | Left TMT (torso-metatarsal) | 3 (ball joint) | None |
| Left shank | 0.456 | Left tarsals | Left ankle | 3 (ball joint) | None |
| Left thigh | 1.183 | Left shank | Left knee | 3 (ball joint) | None |
| Pelvis | 9.047 | Left thigh | Sacro-iliac | 1 (hinge) | Constrained to sagittal plane |
| Lumped torso (spine + head + arms) | 9.448 | Pelvis | None | None | None |
| Right thigh | 1.183 | Left thigh | Right hip | 3 (ball joint) | None |
| Right shank | 0.456 | Right thigh | Right knee | 3 (ball joint) | None |
| Right tarsals | 1.145 | Right shank | Right ankle | 3 (ball joint) | None |
| Right foot | 0.012 | Right tarsals | Right TMT | 3 (ball joint) | None |

Appendix S1: Converting joint kinematics to quaternions (see Richards & Porro, 2018 for further details).

Briefly, a unit quaternion (*q*) is a 4D unit vector encoding a 3D rotation axis and an angle about that axis. Analogous to a rotation matrix (Cartesian reference frame), a *q* encodes 3D spatial orientation whilst avoiding the numerical pitfalls of Euler angles, hence are ideal for modelling. Disregarding long-axis orientation, each *i^th^* body segment (for a total of *ns* segments) on the left leg and torso is represented as a vector:

| $\mathbf{V}=\left[ \mathbf{v}_{\mathbf{i,i+1}}\boldsymbol{\ldots},\mathbf{v}_{\mathbf{ns-1, ns}} \right]=[\mathbf{p}_{\mathbf{i+1}}-\mathbf{p}_{\mathbf{i}}\ldots,\mathbf{p}_{\mathbf{ns-1}}-\mathbf{p}_{\mathbf{ns}}]$ | (S1.1) |
| --- | --- |

Each vector is used to calculate an axis of rotation between adjacent segments. Note that it is a unit vector.

| $\mathbf{a}_{\boldsymbol{i}}\boldsymbol{=}\frac{\mathbf{v}_{\boldsymbol{ref}}\boldsymbol{\times}\mathbf{v[}i\boldsymbol{]}}{\left\Vert\mathbf{v}_{\boldsymbol{ref}}\boldsymbol{\times}\mathbf{v[}i\boldsymbol{]} \right\Vert}$ | (S1.2) |
| --- | --- |

where **V_ref_** is a reference vector (e.g. [0, 0, 1]). Then the angle between adjacent segments is calculated:

| $\theta= {cos}^{-1}\frac{\mathbf{v1}\cdot\mathbf{v2}}{\left\Vert\mathbf{v1} \right\Vert\left\Vert\mathbf{v2} \right\Vert}$ | (S1.3) |
| --- | --- |

Where **V_1_** and **V_2_** are adjacent segment vectors. The axis and angle are used to compose a unit quaternion representing segment orientation (relative to the adjacent segment);

| $\mathbf{q}=[\cos\left( \frac{\theta}{2} \right),\hat{a_{x}}\sin\left( \frac{\theta}{2} \right),\hat{a_{y}}\sin\left( \frac{\theta}{2} \right),\hat{a_{z}}\sin\left( \frac{\theta}{2} \right) ]$ | (S1.4) |
| --- | --- |

Where **a**_x,y,z_ are the components of the rotation axis (Eq. S1.2) expressed as a unit vector and θ is the angle in radians (Eq. S1.3). Finally, the quaternions are assembled into a vector:

| $\mathbf{Q}=\left[ \mathbf{q}_{\mathbf{i,i+1}}\boldsymbol{\ldots},\mathbf{q}_{\mathbf{ns-1, ns}} \right]= \left[ \mathbf{q}_{\mathbf{torso}}\boldsymbol{\ldots},\mathbf{q}_{\mathbf{prox.foot}} \right]$ | (S1.5) |
| --- | --- |

Segment kinematics, expressed as unit quaternions, were smoothed by first converting them to Hopf coordinates (to preserve quaternion unity), applying a 4^th^ order polynomial fit, then converting back to unit quaternions (SI).

Richards, Christopher T., and Laura B. Porro. "A novel kinematics analysis method using quaternion interpolation–a case study in frog jumping." *Journal of theoretical biology* (2018).

Appendix S2: Time-varying function for IS extension

The IS angle (*θ_IS_*) was increased or decreased through time to mimic the action of pelvic musculature.

| $\theta_{IS}=0.5\tanh\left[ 2\pi a\left( 2ts-2b \right) \right]+0.5$  $0\leq ts\leq36$ | (S2.1) |
| --- | --- |

This defined a rounded step function for *θ_IS_* (in radians) of simulation time, *ts*, rising from 0 to 1 at a steepness determined by *a* and a rise onset time of *b* (*a* = 0.01 for non-linear versus *a* = 0.001 for ~linear; *b* = 30 for the current study; Fig. S1&2). The time units, *ts*, are scaled to the sample frequency of the example jump used from experimental data. To convert to absolute time, we multiply by the data capture sample interval (*t* = *ts**0.004 s). For small values of *a*, (*a* ~0.001) the function rises approximately linearly whereas the curve is a sigmoid approaching a step at increasing values.

Appendix S3: Inverse Dynamics with simulated contacts (IDC)

Joint torques and ground reaction forces (GRF) were calculated by inputting kinematics into MuJoCo’s soft contact solver to solve for joint-space generalised forces, **τ**:

| **τ** = **Ma**+**c + g** −**Jc^T^f** | (S3.1) |
| --- | --- |

Where **M** and **Jc** are the mass-inertia and constraint jacobian matrices and **Ma**, **c, g**, **J^T^f** are the components of torque required to overcome limb inertia, Coriolis, gravity and constraint forces (including GRF), respectively, to cause joint acceleration, **a**.

SUPPLEMENTARY FIGURES & MOVIES


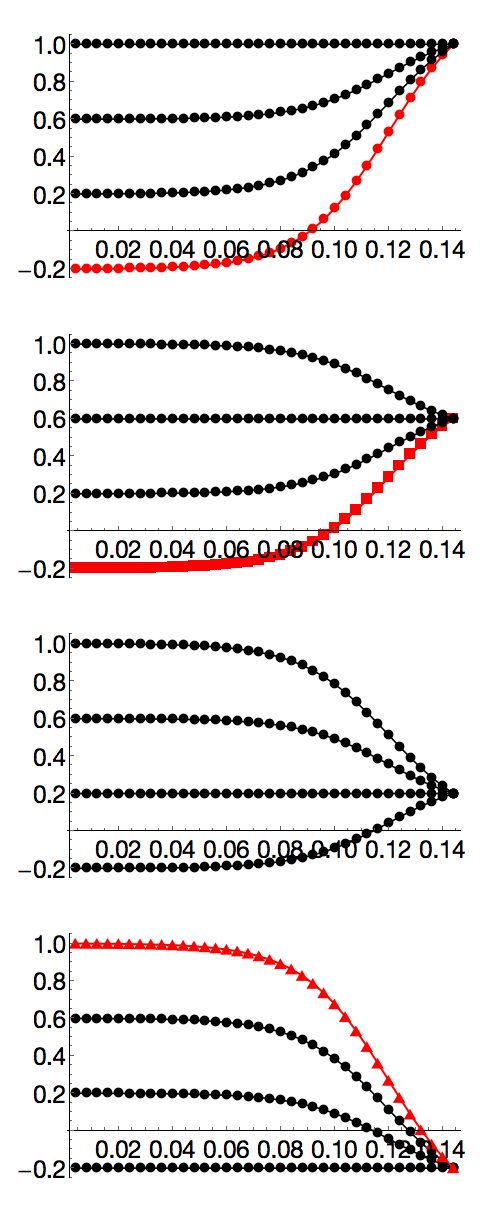


Figure S1. Simulated non-linear ilio-sacral (IS) angle traces for 16 simulations. IS profiles are defined by Eq. S1 with the steepness parameter, a = 0.01. Increasing initial angles (lower values = closer to ground) of -0.2, 0.2, 0.6, and 1.0 are iterated for each set for final IS angle values of -0.2, 0.2, 0.6, and 1.0 for panels A-D, respectively. Positive slope represent IS joint extension. All values are in radians to be consistent with the Eq. S1. The three exemplar simulations shown in Fig. 1 are sim1) the steepest (circle), sim2) the farthest (square) and sim3) the shortest-lowest (triangle).


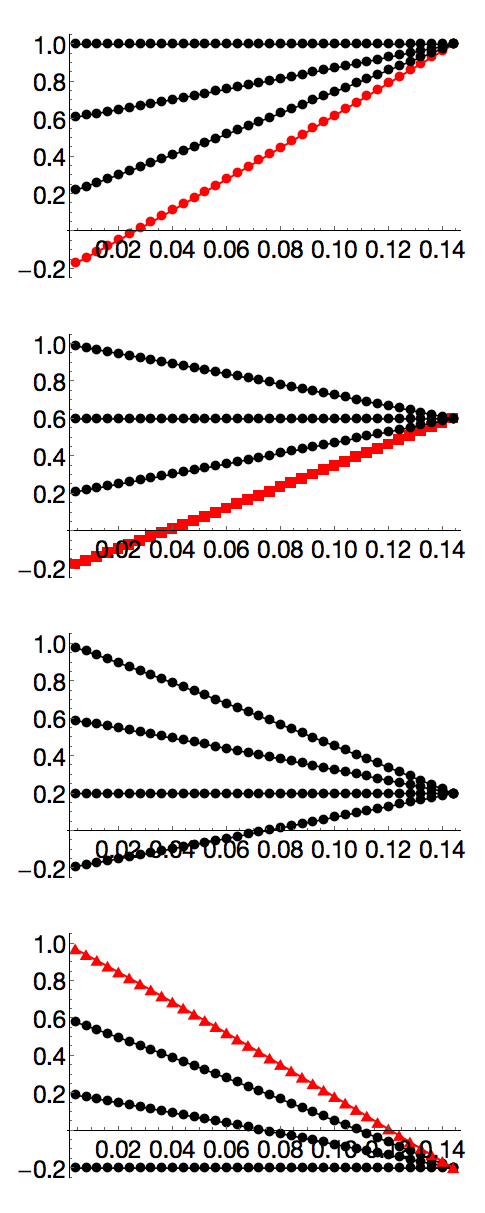


Figure S2. Simulated linear ilio-sacral (IS) angle traces for 16 simulations. IS profiles are defined by Eq. S1 with the steepness parameter, *a* = 0.001 for approximate linearity. Increasing initial angles (lower values = closer to ground) of -0.2, 0.2, 0.6, and 1.0 are iterated for each set for final IS angle values of -0.2, 0.2, 0.6, and 1.0 for panels A-D, respectively. Positive slope represent IS joint extension. All values are in radians to be consistent with the Eq. S1. The three exemplar simulations shown in Fig. 1 are sim1) the steepest (circle), sim2) the farthest (square) and sim3) the shortest-lowest (triangle).

SI movie 1: Example simulation (sim 1) showing the steepest jump in three views. The red ball indicates the time-varying position of the centre of mass. The simulated ground reaction force vector (left leg only) is shown in black. The simulation is paused at the moment of takeoff.

SI movie 2: Example simulation (sim 2) showing the farthest jump in three views. The red ball indicates the time-varying position of the centre of mass. The simulated ground reaction force vector (left leg only) is shown in black. The simulation is paused at the moment of takeoff.

SI movie 3: Example simulation (sim 3) showing the shortest (most horizontal) jump in three views. Unlike sim 1&2, the torso rotates downward to cause a more horizontal takeoff trajectory. The red ball indicates the time-varying position of the centre of mass. The simulated ground reaction force vector (left leg only) is shown in black. The simulation is paused at the moment of takeoff.

SI movie 4: Four example simulations where slight changes in the timing and magnitude of IS extension influences the duration of ground contact, even with identical leg kinematics. Simulation time is shown in seconds. Because the simulation ends automatically when vertical force crosses zero, any alterations of GRF due to IS extension can dramatically alter ground contact time to cause a premature takeoff. The simulated ground reaction force vector (left leg only) is shown in black. The red ball indicates the time-varying position of the centre of mass. The simulation is paused at the moment of takeoff. Note that there is some error (+/- one time step) in our algorithm’s estimation of vertical force zero crossing, causing the final animation frame to either show positive values (one frame prior to takeoff) or negative values (one frame after takeoff).

SI movie 5: Three example simulations showing increasing, but fixed IS angles (representing the lower-left to upper-right diagonal of the IS angle parameter space (see Fig. 2). The red ball indicates the time-varying position of the centre of mass. The simulated ground reaction force vector (left leg only) is shown in black. The simulation is paused at the moment of takeoff.
